# Statistical analysis plan for the Dex-CSDH trial: a randomised, double-blind, placebo-controlled trial of a 2-week course of dexamethasone for adult patients with a symptomatic chronic subdural haematoma

**DOI:** 10.1186/s13063-019-3866-6

**Published:** 2019-12-10

**Authors:** Annabel Allison, Ellie Edlmann, Angelos G. Kolias, Carol Davis-Wilkie, Harry Mee, Eric P. Thelin, Carole Turner, Peter J. Hutchinson, Simon Bond

**Affiliations:** 1 Cambridge Clinical Trials Unit, Coton House Level 6, Cambridge University Hospitals NHS Foundation Trust, Hills Road, Cambridge, CB2 0QQ UK; 2 University of Cambridge & Cambridge University Hospitals NHS Foundation Trust, Division of Neurosurgery, Box 167, University of Cambridge, Cambridge Biomedical Campus, Cambridge, CB2 0QQ UK; 3 SouthWest Neurosurgical Centre, University Plymouth Hospitals NHS trust, Plymouth, PL6 8DH UK; 4 Department of Clinical Neuroscience, Karolinska Institutet, Stockholm, Sweden; 5 Theme Neuro, Karolinska University Hospital, Stockholm, Sweden

**Keywords:** Dexamethasone, Chronic subdural haematoma, Randomised trial, Steroid

## Abstract

**Background:**

The incidence of chronic subdural haematoma (CSDH) is increasing. Although surgery remains the mainstay of management for symptomatic patients, uncertainty remains regarding the role of steroids. Hence, the Dex-CSDH trial was launched in the UK in 2015 aiming to determine whether, compared to placebo, dexamethasone can improve the 6-month functional outcome of patients with symptomatic CSDH by reducing the rate of surgical intervention and recurrence rate.

**Methods and design:**

Dex-CSDH is a multi-centre, pragmatic, parallel group, double-blind, randomised trial assessing the clinical utility of a 2-week course of dexamethasone following a CSDH. Seven hundred fifty patients were randomised to either dexamethasone or placebo. The primary outcome is the modified Rankin Scale at 6 months which is dichotomised to favourable (a score of 0–3) versus unfavourable (a score of 4–6).

**Conclusions:**

This paper and the accompanying additional material describe the statistical analysis plan for the trial.

**Trial registration:**

ISRCTN, ISRCTN80782810. Registered on 7 November 2014. http://www.isrctn.com/ISRCTN80782810. EudraCT, 2014-004948-35. Registered on 20 March 2015.

## Background

Chronic subdural haematoma (CSDH) is a collection of blood overlying the surface of the brain which is diagnosed on cranial imaging as a predominantly hypodense or isodense collection in the subdural space on computed tomography [1]. It is especially common in older patients and can happen with only a minor injury to the head or even in the absence of trauma. Symptoms that can be attributed to a CSDH include headache, gait disturbance, falls, confusion, focal neurological deficit, speech disturbance, drowsiness, and seizures.

The incidence of CSDH is increasing [2]. In the UK, approximately 5000 people aged over 65 years are diagnosed with a CSDH each year. In the National Health Service (NHS), patients who are symptomatic usually have an operation to evacuate the CSDH. Patients with very mild symptoms or small collections are usually actively monitored. Although about 80% of patients tend to recover well from surgery, up to 20% of patients will have a recurrence of the CSDH requiring further surgery. This significantly reduces the chances of a good recovery.

One theory on the formation of a CSDH is that, following a traumatic injury, an inflammatory reaction is initiated which drives the growth of abnormal blood vessels and fluid accumulation over the brain surface, supported by findings of high levels of inflammatory markers within the CSDH fluid [3–6]. Steroids, such as dexamethasone, are thought to help dampen this inflammatory reaction, allowing better resolution of the CSDH and lower likelihood of recurrence. The Dex-CSDH (DEXamethasone in Chronic SubDural Haematoma) trial will determine whether steroids should be prescribed routinely for patients with a symptomatic CSDH.

## Methods and design

Dex-CSDH is a multi-centre, clinical phase III, randomised, double-blind, placebo-controlled trial of dexamethasone for up to 2 weeks on clinical outcome following CSDH. In total, 750 symptomatic patients with a radiological diagnosis of CSDH, recruited from neurosurgical units (NSUs) based within the UK, were randomised to a 2-week tapering course of either dexamethasone or placebo stratified by site. The primary outcome measure was the modified Rankin Scale (mRS) at 6 months (dichotomised to 0–3 and 4–6, as used in previous studies [7, 8]), which was adapted from a validated questionnaire [9]. The decision to dichotomise the mRS was based on the clinical interpretability of the outcome and its potential value in future meta-analyses. Secondary outcome measures sought a difference in the acute clinical course, mortality, length of stay in hospital, discharge destination, and adverse events and complications. All research staff and outcome assessors were blinded. Full details of the study design can be found in the protocol [10].

A sample size re-estimation was performed, using data on 450 patients, due to uncertainty in the absolute favourable outcome rate of the primary outcome (score of 0–3) in the control arm. The possible adaptations were to either increase the sample size (up to a maximum of 1000) or to stop the trial for futility if the revised sample size was >1000; the original sample size would be retained if the re-estimated sample size was <750. An Independent Data Monitoring and Ethics Committee (IDMEC) reviewed the sample size re-estimation and recommended the trial continue to recruit to the original target of 750 patients. The investigators remained blinded to the analysis.

The statistical analysis plan (SAP) was written prior to final analysis and unblinding in order to avoid reporting bias and data-driven results. The final outcomes will be collected by July 2019, with the unblinded analysis expected to take place in early 2020. The following sections outline the main analyses featured in the SAP. Full details are provided in Additional file 1.

### Subject disposition

A Consolidated Standards of Reporting Trials (CONSORT) diagram will be produced to show patient disposition in accordance with the CONSORT statement [11]. A mock CONSORT diagram is provided in Additional file 1.

### Demographic and baseline variables

Summary statistics for a range of demographic and baseline variables (health status, injury background, surgical procedures, blood products given, concurrent illnesses, prior and concomitant medications) will be produced. Continuous variables will be summarised using the following descriptive statistics: *n* (non-missing sample size), mean, standard deviation, median, maximum, and minimum. The frequency and percentages (based on the non-missing sample size) of observed levels will be reported for all categorical measures.

### Primary efficacy analyses

The primary analysis will estimate the absolute difference between the two treatment arms in the proportions achieving a favourable outcome (mRS of 0–3 at 6 months). A normal approximation will be used to produce a 95% confidence interval and two-sided *P* value to test the confirmatory hypothesis that there is no difference in the primary outcome between the two treatment arms versus the alternative that there is a difference. The study was adequately powered for this choice of analysis, which makes as few assumptions as possible.

As a secondary analysis, a proportional odds logistic regression model will be fitted to the original mRS score, adjusting for the baseline covariates age and Glasgow Coma Scale (GCS) [12]. The model will not adjust for the randomisation strata site, as the main reason for stratification was investigational medicinal product (IMP) management—to enable sites to keep a balanced supply of drugs whilst maintaining the blinding. This will not significantly influence the outcome; the treatment was given by a protocolised dosing regimen, so practice did not differ between sites.

A number of exploratory subgroup analyses will be undertaken by fitting logistic regression models to the primary outcome and looking for a treatment interaction effect with the following subgroups: site, age, timing of head trauma, use of anticoagulants or anti-platelets, GCS score at baseline, and unilateral versus bilateral CSDH.

Subjects will be analysed as randomised using both a full analysis population (all randomised subjects excluding those randomised in error) and a per protocol population. The per protocol population will include subjects who take at least 80% of their medication and exclude subjects in the placebo group who receive >8mg of dexamethasone during the treatment course. The former condition will be based on remaining pill count at the end of the treatment period; the latter will be based on concomitant medication information, which may not be recorded accurately.

The effect of treatment compliance on the primary outcome will also be investigated in an exploratory manner, including using an instrumental variables approach to estimate the effect of compliance, measured as a continuous percentage of medication taken.

### Missing data

The sample size of non-missing values will be reported for summary tables. For the primary and secondary outcomes a graph showing the percentage of missing data will be produced.

Patients who die during the conduct of the study will receive an outcome score of “6-Dead” for all mRS assessments occurring after the date of death. If there is less than 15% missing data for the primary outcome, then a complete case analysis will be used, which assumes data are missing completely at random. If there is more than 15% missing data for the primary outcome, then a sensitivity analysis will be performed using the method described in [13].

### Secondary efficacy analyses

The secondary outcomes are:

- Number of CSDH-related surgical interventions undertaken during the primary admission
- Number of CSDH-related surgical interventions undertaken during subsequent admissions
- GCS at discharge and at 6 months (categorised as 0 to 7, 8 to 12, and 13 to 15)
- mRS at discharge and at 3 months
- Barthel Index [14] at discharge, 3 months, and 6 months
- European Quality of Life five-dimension, five-level scale (EQ-5D-5L) [15–17] at discharge, 3 months, and 6 months
- All-cause mortality at 30 days and 6 months
- Discharge information: length of stay in NSU, length of stay in secondary care, discharge destination.

The number of surgical interventions undertaken during the primary admission will be defined in two ways and the analysis repeated for each: (1) including pre-randomisation surgical procedures which occur within the 72 h prior to randomisation, and (2) excluding pre-randomisation surgical procedures. The clinical reason for including pre-randomisation procedures is that the exact timing of the first dose in relation to surgery is thought to have little bearing on the pathophysiology; rather it is the 2-week course of treatment that will have the greatest impact. However, from a statistical point of view it is preferable to exclude pre-randomisation procedures from the outcome, as any differences in outcome may simply be due to differences in pre-randomisation procedures, and so this will be performed as a secondary analysis.

The effect of treatment on each outcome will be modelled using Poisson regression for count data (number of CSDH surgical interventions, length of stay), proportional odds logistic regression for ordinal data (GCS, mRS), linear regression for continuous data (Barthel Index, EQ-5D-5L), and logistic regression for categorical data (discharge destination). All models will be unadjusted for any baseline covariates. Length-of-stay variables are calculated as the total length of stay across all admissions and will be treated as discrete counts (measured in days), and therefore analysed using Poisson regression.

### Post-randomisation subgroup analyses

As exploratory analyses, summary statistics (frequency and percentage for the primary outcome) will be produced by treatment group for each of the following post-randomisation subgroups:

- Recurrence versus non-recurrence of the CSDH
- Conservative management versus non-conservative management (no surgery on any admission versus ≥1 operation)
- Trial conservative management (surgery within 7 days of randomisation versus surgery more than 7 days after randomisation versus no surgery at any time point)
- Surgical intervention during primary surgery (burr hole, mini-craniotomy)
- Drain during primary surgery versus no drain during primary surgery.

Here recurrence is defined as a symptomatic recurrence requiring re-operation of a previously evacuated ipsilateral CSDH.

The recurrence rate is reported in the majority of surgical CSDH studies, and therefore its calculation will allow cross-study comparisons [18].

A mediation analysis will be performed to look at both the direct effect of treatment on the primary outcome and the indirect effect of treatment via the mediator variable recurrent CSDH, by estimating the causal parameters using parametric regression models for the mediator and outcome. We will assume there are no unmeasured confounders, and therefore results may be biased.

### Safety analyses

Listings of adverse events (AEs) and serious adverse events (SAEs) will be produced and will include information on the following: the event, the onset and resolution dates of the event, Medical Dictionary for Regulatory Activities (MedDRA) preferred term, MedDRA system organ class (SOC), causality, and outcome. The SAE listing will also include information on the severity and seriousness of the event.

The frequency and percentage of MedDRA SOC codes will be reported. Plots showing the incidence of AEs and SAEs and their relative risk (with 95% confidence interval) based on the MedDRA SOC codes will also be produced.

### Health economics

A health economics analysis is planned but is documented in a separate health economics analysis plan.

### Supporting information

Additional file 1 provides the full SAP.

### Trial status

At the time of first submission, the Dex-CSDH trial had finished recruiting the target of 750 patients and was in follow-up.

## Supplementary information

**Additional file 1** Statistical analysis plan: final analysis

## Data Availability

Research data underpinning published research findings will be deposited in the University of Cambridge repository. The Trial Management Group will be responsible for providing access to research data requested by third parties as freely and timely as possible, unless access to the data is restricted by a legal obligation (e.g. non-disclosure agreement), intellectual property protection, ethical approval requirements, ethical or security reasons, or other legitimate reasons (such reasons will be stated in the metadata description).

## Abbreviations

AE: Adverse event
CSDH: Chronic subdural haematoma
GCS: Glasgow Coma Scale
IMP: Investigational medicinal product
MedDRA: Medical Dictionary for Regulatory Activities
mRS: Modified Rankin Scale
NSU: Neurosurgical unit
SAE: Serious adverse event
SAP: Statistical analysis plan
SOC: System organ class
