# Supplementary material for: Statistical analysis plan for the Dex-CSDH trial: a randomised, double-blind, placebo-controlled trial of a 2-week course of dexamethasone for adult patients with a symptomatic chronic subdural haematoma
Source: Trials. 2019 Dec 10;20:698. doi: 10.1186/s13063-019-3866-6 (PMC6905057; doi:10.1186/s13063-019-3866-6)
Supplement: Supplementary file 1 — Additional file 1 Statistical analysis plan: final analysis [file 13063_2019_3866_MOESM1_ESM.docx]

CCTU/TPL007V3 Approved:24/01/2018

Statistical Analysis Plan - Final Analysis

| TRIAL FULL TITLE | A randomised, double blind, placebo-controlled trial of a two-week course of dexamethasone for adult patients with a symptomatic Chronic Subdural Haematoma (Dex-CSDH trial) |
| --- | --- |
| EUDRACT NUMBER | 2014-004948-35 |
| SAP VERSION | 0.12 |
| ISRCTN NUMBER | 80782810 |
| SAP VERSION DATE | 14/10/2019 |
| TRIAL STATISTICIAN | Dr Simon Bond |
| TRIAL CHIEF INVESTIGATOR | Professor Peter Hutchinson |
| SAP AUTHOR | Annabel Allison |

# SAP Signatures

I give my approval for the attached SAP entitled Dex-CSDH dated 14 October 2019.

**Chief Investigator**

Name: Professor Peter Hutchinson

Signature:                                                

Date:

**Trial Statistician**

Name: Dr Simon Bond

Signature:                                                

Date:

**SAP Author**

Name: Annabel Allison

Signature:                                                

Date:

# Table of Contents

[1 SAP Signatures 1](#_Toc2332340)

[2 Table of Contents 3](#_Toc2332341)

[3 Abbreviations and Definitions 6](#_Toc2332342)

[4 Introduction 7](#_Toc2332343)

[4.1 Preface 7](#_Toc2332344)

[4.2 Purpose of the Analyses 7](#_Toc2332345)

[5 Study Objectives and Endpoints 8](#_Toc2332346)

[5.1 Study Objectives 8](#_Toc2332347)

[5.1.1 Primary Objective 8](#_Toc2332348)

[5.1.2 Secondary Objectives 8](#_Toc2332349)

[5.1.3 Exploratory Objectives (sub-study) 8](#_Toc2332350)

[5.2 Endpoints 8](#_Toc2332351)

[5.2.1 Primary 8](#_Toc2332352)

[5.2.2 Secondary 8](#_Toc2332353)

[5.2.3 Exploratory 9](#_Toc2332354)

[6 Study Methods 9](#_Toc2332355)

[6.1 General Study Design and Plan 9](#_Toc2332356)

[6.2 Inclusion-Exclusion Criteria and General Study Population 9](#_Toc2332357)

[6.2.1 Inclusion Criteria 9](#_Toc2332358)

[6.2.2 Exclusion Criteria 10](#_Toc2332359)

[6.2.3 Exclusion Criteria for Biochemical Sub-study 10](#_Toc2332360)

[6.2.4 Exclusion Criteria for MRI Sub-study 10](#_Toc2332361)

[6.3 Randomisation and Blinding 12](#_Toc2332362)

[6.3.1 Randomisation 12](#_Toc2332363)

[6.3.2 Blinding 12](#_Toc2332364)

[6.4 Study Variables 13](#_Toc2332365)

[6.4.1 Modified Rankin Scale 14](#_Toc2332366)

[6.4.2 EuroQoL EQ-5D-5L 14](#_Toc2332367)

[6.4.3 Glasgow Coma Scale 15](#_Toc2332368)

[6.4.4 Barthel Index 15](#_Toc2332369)

[7 Sample Size 18](#_Toc2332370)

[8 General Considerations 18](#_Toc2332371)

[8.1 Timing of Analyses 18](#_Toc2332372)

[8.2 Analysis Populations 19](#_Toc2332373)

[8.2.1 Full Analysis Population 19](#_Toc2332374)

[8.2.2 Per Protocol Population 19](#_Toc2332375)

[8.2.3 Safety Population 19](#_Toc2332376)

[8.3 Covariates and Subgroups 19](#_Toc2332377)

[8.3.1 Post-randomisation subgroups 20](#_Toc2332378)

[8.4 Missing Data 21](#_Toc2332379)

[8.5 Interim Analyses and Data Monitoring 21](#_Toc2332380)

[8.6 Multi-centre Studies 22](#_Toc2332381)

[8.7 Multiple Testing 22](#_Toc2332382)

[9 Summary of Study Data 22](#_Toc2332383)

[9.1 Subject Disposition 22](#_Toc2332384)

[9.2 Derived variables 24](#_Toc2332385)

[9.3 Protocol Deviations 25](#_Toc2332386)

[9.4 Demographic and Baseline Variables 25](#_Toc2332387)

[9.4.1 Demographics 25](#_Toc2332388)

[9.4.2 Health Status Prior to CSDH 26](#_Toc2332389)

[9.4.3 Injury background 26](#_Toc2332390)

[9.4.4 Imaging, Surgery, and Complications 26](#_Toc2332391)

[9.4.5 Treatments Given 27](#_Toc2332392)

[9.5 Concurrent Illnesses and Medical Conditions 27](#_Toc2332393)

[9.6 Prior and Concurrent Medications 28](#_Toc2332394)

[9.7 Treatment Compliance 28](#_Toc2332395)

[9.8 Withdrawals 28](#_Toc2332396)

[10 Efficacy Analyses 29](#_Toc2332397)

[10.1 Primary Efficacy Analysis 29](#_Toc2332398)

[10.2 Secondary Efficacy Analyses 31](#_Toc2332399)

[10.2.1 Surgery 31](#_Toc2332400)

[10.2.2 Questionnaires 31](#_Toc2332401)

[10.2.3 Mortality 32](#_Toc2332402)

[10.2.4 Discharge Information 32](#_Toc2332403)

[10.2.5 Health Economics Analyses 33](#_Toc2332404)

[10.3 Exploratory Efficacy Analyses 33](#_Toc2332405)

[11 Safety Analyses 33](#_Toc2332406)

[11.1 Adverse Events of Special interest 34](#_Toc2332407)

[11.2 Deaths, Serious Adverse Events and other Significant Adverse Events 35](#_Toc2332408)

[11.3 Pregnancies 35](#_Toc2332409)

[12 Figures 35](#_Toc2332410)

[13 Reporting Conventions 36](#_Toc2332411)

[14 Technical Details 36](#_Toc2332412)

[15 References 38](#_Toc2332413)

[16 Listing of Tables, Listings and Figures 40](#_Toc2332414)

# Abbreviations and Definitions

| AE | Adverse Event |
| --- | --- |
| BI | Barthel Index |
| CRF | Case Report Form |
| CSDH | Chronic Subdural Haematoma |
| CSF | Cerebrospinal Fluid |
| CT | Computerised Tomography |
| GCS | Glasgow Coma Scale |
| IDMC | Independent Data Monitoring Committee |
| IMP | Investigational Medical Product |
| IWRS | Interactive Web-based Response System |
| MHRA | Medicines and Healthcare products Regulatory Agency |
| MRI | Magnetic Resonance Imaging |
| mRS | modified Rankin Scale |
| NG | Nasogastric |
| NHS | National Health Service |
| NSU | Neurosurgical Unit |
| SAP | Statistical Analysis Plan |
| TCD | Transcranial Doppler |

#

# Introduction

## Preface

Chronic subdural haematoma (CSDH) is defined as a predominantly hypodense or isodense collection in the subdural space along the cerebral convexity on computerised tomography (CT). It is especially common in older patients and can happen with only a minor injury to the head even in the absence of trauma. Symptoms that can be attributed to a CSDH include headache, gait disturbance, falls, confusion/cognitive decline, focal neurological deficit, speech disturbance, drowsiness/decreased consciousness, and seizures.

In the UK, 5,000 people aged over 65 years are diagnosed with a CSDH each year. In the National Health Service (NHS), patients with severe symptoms usually undergo an operation to evacuate the CSDH. Patients with milder symptoms are usually actively monitored. Although about 80% of the patients tend to recover well from this operation, up to 20% of patients will have a recurrence of the CSDH and require a further operation. This significantly reduces the chances of recovery.

Dex-CSDH (**DEX**amethasone in **C**hronic **S**ub**D**ural **H**aematoma) is a multi-centre, clinical phase III, randomised, double blind, placebo controlled trial of dexamethasone for up to 2 weeks, on clinical outcome following CSDH. 750 patients with a clinical and radiological diagnosis of CSDH will be recruited from neurosurgical units based within the UK. The primary outcome measure is the modified Rankin Scale (mRS) at 6 months; secondary outcome measures seek differences in the acute clinical course, mortality, length of stay in hospital, discharge destination, and adverse events (AEs) and complications.

## Purpose of the Analyses

These analyses will evaluate the efficacy and safety of a two-week course of dexamethasone in comparison with placebo for adult patients with a symptomatic CSDH.

# Study Objectives and Endpoints

## Study Objectives

(ICH E3; 8)

### Primary Objective

- To determine the clinical effectiveness of a two-week course of dexamethasone for adult patients with a symptomatic CSDH

### Secondary Objectives

- Compare the AEs and complications between the two arms
- Undertake a detailed economic evaluation

### Exploratory Objectives (sub-study)

- Assess the biological action of dexamethasone within the CSDH
- Assess the role of dexamethasone in cerebral perfusion and oedema in CSDH

## Endpoints

(ICH E9; 2.2.2)

### Primary

- mRS at 6-months which is dichotomised to favourable (0-3) vs unfavourable (4-6)

### Secondary

- Number of CSDH-related surgical interventions undertaken during the index admission
- Number of CSDH-related surgical interventions undertaken during the subsequent admission in the follow-up period
- Glasgow Coma Scale (GCS) at discharge from Neurosurgical Unit (NSU) and at 6-months
- mRS at discharge from NSU and at 3-months
- Barthel Index (BI) at discharge from NSU, 3-months, and 6-months
- Mortality at 30 days and 6-months
- EuroQol EQ-5D at discharge from NSU, 3-months, and 6-months
- Length of stay in NSU
- Discharge destination from NSU
- Length of stay in secondary care
- Health-economic analysis
- AEs

### Tertiary

- Recurrence (defined as a symptomatic recurrence requiring re-operation of a previously evacuated ipsilateral CSDH)

### Exploratory

- Assessment of inflammatory mediators in CSDH fluid and blood
- Cerebral perfusion measured using Magnetic Resonance Imaging (MRI) and Transcranial Doppler (TCD)
- Cerebral swelling measured using MRI

# Study Methods

## General Study Design and Plan

(ICH E3; 9)

The study is a pragmatic, multi-centre, parallel group, double-blind, phase III, randomised, placebo-controlled trial with a sample size re-estimation assessing the clinical utility of a tapering 2-week course of dexamethasone following CSDH. Treatment will be assigned using permuted block randomisation. Figure 1 shows the timing of randomisation and follow-up visits.

## Inclusion-Exclusion Criteria and General Study Population

(ICH E3; 9.3. ICH E9; 2.2.1)

### Inclusion Criteria

- Adult patients aged 18 years or older
- Symptomatic CSDH confirmed on cranial imaging (e.g. CT/MRI - predominantly hypodense or isodense crescentic collection along the cerebral convexity on CT)
- Patient or Legal Representative is willing and able to provide informed consent or in the absence of a legal representative, an Independent Healthcare Professional provides authorisation for patient enrolment

### Exclusion Criteria

The presence of any of the following will preclude patient inclusion:

- Patients with conditions where steroids are clearly contra-indicated
- Patients who are on (or within one month of) regular oral or intravenous glucocorticoid steroids^[[1]](#footnote-1)^
- Previous enrolment in this trial for a prior episode
- Time interval from the time of admission to NSU to first dose of trial medication exceeds 72 hours
- CSDH in the presence of a cerebrospinal fluid (CSF) shunt
- Severe lactose intolerance or any known hypersensitivity to dexamethasone or any of the investigational medicinal product (IMP) excipients
- Patients with a previous history of psychotic disorders
- Unwillingness to take products containing gelatine
- Concurrent enrolment in any other trial of an IMP

### Exclusion Criteria for Biochemical Sub-study

Patients will be excluded from recruitment to the biochemical part of the study if they have any of the following:

- Active malignancy
- On immunosuppressive drug therapy

### Exclusion Criteria for MRI Sub-study

Patients will be excluded from recruitment to the MRI part of the study if they have any of the following:

- Renal dysfunction
- Pacemaker or any metal implants


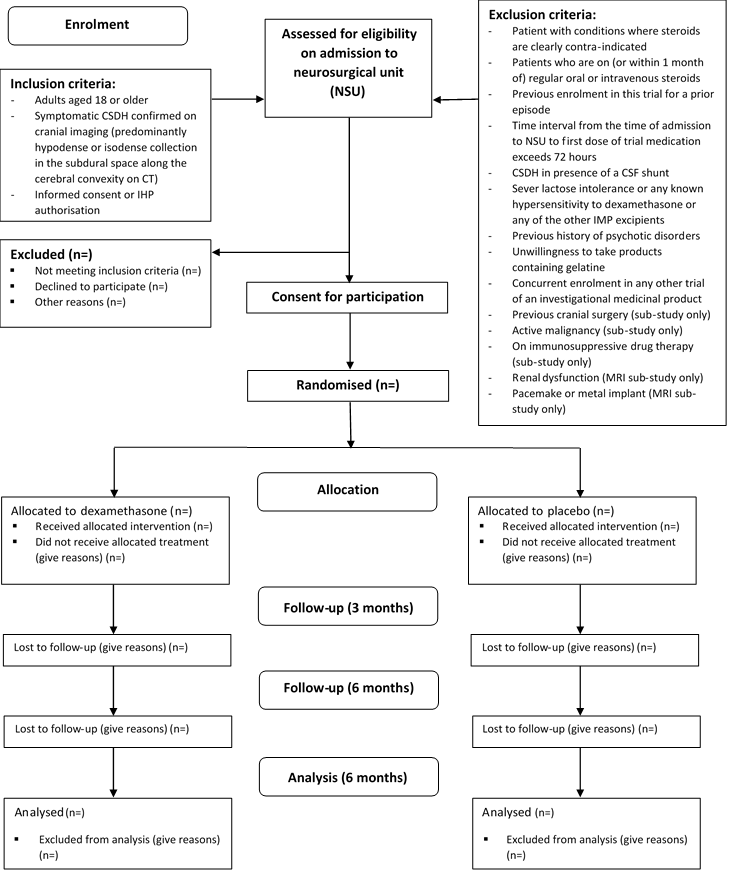


Figure 1: Study design flowchart

## Randomisation and Blinding

(ICH E3; 9.4.3, 9.4.6. ICH E9; 2.3.1, 2.3.2)

### Randomisation

Patients will be randomly assigned to either the control or intervention group with a 1:1 allocation as per a computer generated randomisation schedule stratified by site using permuted blocks of random sizes. An Interactive Web-based Response System (IWRS) will be used for allocating treatment packs to individual patients once confirmation of inclusion criteria being met is confirmed.

### Blinding

Capsules and packaging for both active and placebo arms will be identical in appearance at the point of issue to patients.

It is estimated that less than 10% of eligible patients will have (or develop during the trial) swallowing difficulties, making oral IMP administration difficult or impossible/unsafe. To ensure the trial can proceed in as representative a population as possible, a pragmatic and cost-effective approach to dosing IMP in this cohort is proposed.

The strategy for managing IMP administration in patients with swallowing difficulties has been developed after discussion and advice from the Medicines and Healthcare products Regulatory Agency (MHRA). The blinded capsules may, with investigator and local pharmacy approval, be opened at the point of administration either via the oral route or a nasogastric (NG) tube if one has been inserted during the routine course of care.

If this scenario occurs, the administering nurse, NHS site pharmacy, and potentially the trial patient will no longer be blinded, because the active dexamethasone is in tablet form that has been over-encapsulated, so may require crushing before dispersal in 15-20ml water for NG administration (administer via a NG tube), and the placebo will be in powder form.

To maintain blinding of the neurosurgeons, the presence of tablets being inside the opened capsule should not be documented in the medical notes, but referred to in generic terms e.g. “Capsule contents were mixed with water for NG administration”.

Every effort must be made to maintain patient blinding when NG administration is used, by the patient not seeing the capsules being opened. Should, despite these efforts, the patient discover their treatment, they should be asked to not disclose their treatment allocation to any of the other medical personnel they interact with, e.g. surgeons, etc. The research staff and outcome assessors will remain blinded.

There are also clinical aspects that could potentially unblind trial team members to treatments allocated.

Patients receiving dexamethasone will be more likely to have higher blood glucose levels compared to those receiving placebo. This may provide an indication – but not proof – that a patient is in the active arm. Concealment of glucose measurements will be difficult as clinical action may be required. However, any decision about surgery is made based on the severity of symptoms and/or progression of symptoms. It is not conceivable that a hint that a patient is in the active arm would influence any decisions about operative or non-operative management, especially because the use of steroids is limited in the UK. Overall, we believe that blinding of the clinical staff (neurosurgeons) making decisions about surgery will be maintained throughout the trial.

The trial statistician performing the analysis will be blinded to treatment allocation until Version 2.0 of the statistical analysis plan (SAP) has been approved and the database has been hardlocked.

## Study Variables

(ICH E3; 9.5.1. ICH E9; 2.2.2)

Table 1 shows the frequency and timing of study assessments including assessment time-windows. The discharge from NSU, day 15, and day 30 assessment time-windows are all ±1 week; the 3 month and 6 month assessment time-windows are -4/+8 weeks. Subjects whose measurements fall outside the defined time-windows will be excluded from the per protocol analysis.

### Modified Rankin Scale

The mRS (adapted from [1]) measures the degree of disability or dependence in the daily activities in people who have suffered a neurological disability. The score ranges from 0 to 6:

- 0 – No symptoms
- 1 – No significant disability despite symptoms; able to carry out all usual duties and activities
- 2 – Slight disability; unable to carry out previous activities, but able to look after own affairs without assistance
- 3 – Moderate disability; requiring some help, but able to walk without assistance
- 4 – Moderately severe disability; unable to walk without assistance and unable to attend to own bodily needs without assistance
- 5 – Severe disability; bedridden, incontinent and requiring constant nursing care and attention
- 6 – Dead

### EuroQoL EQ-5D-5L

The EuroQoL EQ-5D-5L questionnaire is a validated, self-reported outcome [2] consisting of:

- A visual analogue scale (VAS) where respondents mark their health status on a scale of 0 (worst imaginable health) to 100 (best imaginable health)
- A health status instrument which examines five domains related to daily activities
  - Mobility
  - Self-care
  - Usual activities
  - Pain and discomfort
  - Anxiety and depression

The responses will be converted to an overall score using a published utility algorithm for the UK population [3], in accordance with the NICE position statement [4]. Participants who die during the study will be given a score of zero.

### Glasgow Coma Scale

The GCS provides a practical method for assessment of impairment of conscious level in response to defined stimuli [5]. It is comprised of three scores relating to eye opening, verbal response, and motor response. The individual scores are added together to create a total score ranging from 3 (totally unresponsive) to 15 (best response).

### Barthel Index

The BI [6] consists of 10 items that measure a person’s daily functioning. The items relate to:

- Feeding
- Bathing
- Grooming
- Dressing
- Bowels
- Bladder
- Toilet use
- Transfers (bed to chair and back)
- Mobility (on level surface)
- Stairs

The scores from the 10 items are added to give an overall score ranging from 0 (totally dependent) to 100 (completely independent).

Table 1: Timing of assessments

| **Study visit schedule** (per protocol v3.0 dated 27 April 2017) | **Admission** | **Randomisation** *(Preferable, but not essential for this to occur before surgery)* | **Intra-operative**  *(if applicable)* | **Day 1 of trial drug**  *(<72 hours from admission)* | **Day 2-14 of trial drug** | **Discharge from NSU or at death**  *(± 1 week)* | **Day 15**  *(± 1 week)* | **Day 30**  *(± 1 week)* | **3 month follow up**  **(-4/+8 weeks)** | **6 month follow up**  **(-4/+8 weeks)** |
| --- | --- | --- | --- | --- | --- | --- | --- | --- | --- | --- |
| **Eligibility assessment** | **X** |  |  |  |  |  |  |  |  |  |
| **Informed consent** | **X** |  |  |  |  | **X** |  |  |  | **If attends OPA** |
| **Randomisation** |  | **X** |  |  |  |  |  |  |  |  |
| **Part 1 of CRF sent to Trial Coordinating Centre** |  | **X** |  |  |  |  |  |  |  |  |
| **IMP administration** |  |  |  | **X** | **X** |  |  |  |  |  |
| **Review of AEs** |  | **X** |  | **X** | **X** | **X** | **X** | **X** |  |  |
| **Review of concomitant medication** |  |  |  | **X** | **X** |  |  |  |  |  |
| **Telephone call to assess medication diary** |  |  |  |  |  |  | **X** |  |  |  |
| **Completed CRF faxed to Trial Coordinating Centre** |  |  |  |  |  | **X** |  |  |  |  |
| **Review of routine lab results** | **X** |  |  | **X** | **X** | **X** |  |  |  |  |
| **GCS** | **X** |  |  |  |  | **X** |  |  |  | **If attends OPA** |
| **mRS** | **X** |  |  |  |  | **X** |  |  | **X** | **X** |
| **Mortality** |  |  |  |  |  |  |  | **X** |  | **X** |
| **EQ-5D** |  |  |  |  |  | **X** |  |  | **X** | **X** |
| **Barthel Index** |  |  |  |  |  | **X** |  |  | **X** | **X** |
| **Health Service Questionnaire** |  |  |  |  |  |  |  |  |  | **X** |

# Sample Size

(ICH E3; 9.7.2. ICH E9; 3.5)

An 8% increase in the rate of favourable outcome (mRS 0-3) at 6-months is a plausible and clinically important treatment effect [7] [8] [9] [10] [11]. Assuming a favourable outcome rate of 80%-85% in the control group [7] [12] and using a 2-sided test at the 5% significance level, a sample size of 750 patients (allowing for a 15% loss to follow-up) will enable us to detect an 8% absolute difference in the rate of favourable outcome with a power of 80%-92%.

# General Considerations

## Timing of Analyses

The final analysis will occur when 750 patients have completed their six month assessment or have dropped out prior to their six month assessment. Version 1.0 of the SAP will be approved according to the requirements of CCTU/SOP023 before the data download. Permission to lock the database (softlock) will be requested from the chief investigator (CI) using CCTU/FRM094, and the data programmers will deposit the data in a folder to which the trial statistician is given restricted access (following CCTU/SOP057). Logical and graphical checks will be carried out to check that no out of range values are present. An independent statistician may produce an unblinded report at this stage to compare and contrast to the dummy report in order to check for any errors. Data queries will be referred to the data management team for resolution (using CCTU/TPL064). The trial statistician will remain blinded until Version 2.0 of the SAP has been approved and the database has been hardlocked (sign off of FRM097).

## Analysis Populations

(ICH E3; 9.7.1, 11.4.2.5. ICH E9; 5.2)

Assignment of subjects to the analysis populations will be undertaken prior to breaking the blind.

### Full Analysis Population

- All randomised subjects excluding those randomised in error

where “randomised in error” is defined as those subjects that were randomised into the trial but were later found not to meet the inclusion/exclusion criteria. Subjects will be analysed as randomised.

### Per Protocol Population

Separate per protocol populations will be defined for each assessment time-point (discharge, 30 days, 3 months, and 6 months). Subjects will be included if they satisfy the following conditions:

- Were eligible to take part in the study (those randomised in error will be excluded)
- Took at least 80% of their medication (50 tablets) based on remaining pill count at the end of the treatment period
- Completed their assessments within the given time windows as specified in Section 6.4.

Subjects will be excluded if they were randomised to placebo but received >8mg of dexamethasone during the IMP course. This will be based on information on the concomitant medications form and will be determined on a per patient basis by members of the TMG.

### Safety Population

- All randomised subjects excluding those who were randomised in error

## Covariates and Subgroups

(ICH E3; 9.7.1, 11.4.2.1. ICH E9; 5.7)

Although site is a stratification factor in the randomisation, we will not be adjusting for site in the analyses. The main reason for stratification was operational - primarily to deal with drug management - and it is not thought that site will greatly influence the outcome. There are also a number of sites that recruited less than 10 patients, and, whilst this in itself doesn’t preclude adding in a random effect, given that we do not expect site to impact on the outcome we do not feel it is appropriate to adjust for site as either a fixed or random effect.

Exploratory analyses of the primary outcome will look for a treatment interaction effect with the following subgroups defined at baseline:

- Cambridge versus other sites
- Age (<70 versus ≥70 years)
- Head trauma (no head trauma, occurred ≤4 weeks ago, occurred >4 weeks ago, unknown timing)
- Use of anticoagulants or platelets versus none
- GCS score at baseline
- Unilateral versus bilateral CSDH - as defined in imaging findings

Only if the interaction effect is judged to be statistically and clinically significant will the subgroup-specific treatment effect estimates be presented.

A secondary analyses of the primary outcome will include a model for the original mRS score adjusting for age and GCS at baseline.

### Post-randomisation subgroups

As exploratory analyses, summary statistics (frequency and percentage for the primary endpoint) will be produced by treatment group for each of the following post-randomisation subgroups:

- Recurrent CSDH (≥1re-operation versus no re-operations)
- Surgical intervention during primary surgery (burr hole, mini-craniotomy)
- Drain during primary surgery versus no drain during primary surgery
- Conservative management versus non-conservative management (no surgery on any admission versus ≥1 operation)
- Trial conservative management (surgery within 7 days of randomisation versus surgery >7 days after randomisation versus no surgery at any time-point)

A mediation analysis will be performed to look at both the direct effect of treatment on the primary outcome and the indirect effect of treatment via the mediator variable recurrent CSDH, by estimating the causal parameters using parametric regression models for the mediator and outcome. The assumption of no unmeasured confounders will be made and therefore results may be biased.

## Missing Data

(ICH E3; 9.7.1, 11.4.2.2. ICH E9; 5.3. EMA Guideline on Missing Data in Confirmatory Clinical Trials)

The sample size of non-missing values will be reported for summary tables. For the primary and secondary outcomes a graph showing the percentage of missing data will be produced.

Patients who die during the conduct of the study will receive an outcome score of “6 - Dead” for all mRS assessments occurring after the date of death. If there is less than 15% missing data for the primary outcome then a complete case analysis will be used, which assumes data are missing completely at random. If there is more than 15% missing data for the primary outcome then a sensitivity analysis will be performed using the method described in [13].

## Interim Analyses and Data Monitoring

(ICH E3; 9.7.1, 11.4.2.3. ICH E9; 4.1, FDA Feb 2010 “Guidance for Industry Adaptive Design Clinical Trials for Drugs and Biologics”)

The interim analysis estimated the pooled favourable response rate for the primary outcome (mRS of 0-3 at 6 months) using data on 450 patients in order to re-estimate the sample size required to ensure accurate power. The possible adaptations were to either increase the sample size (with a maximum of 1000) or to stop the trial for futility if the revised sample size was >1000. As the trial could only be stopped for futility, it is not necessary to adjust the confidence interval and P-value at the end of the trial. This was confirmed by simulation work which is documented together with further details of the interim analysis in:

## Multi-centre Studies

(ICH E3; 9.7.1, 11.4.2.4. ICH E9; 3.2)

The data from each centre will be combined for the analysis.

## Multiple Testing

(ICH E3; 9.7.1, 11.4.2.5. ICH E9; 2.2.5)

As there is only one primary outcome, adjustment for multiple testing is not required.

# Summary of Study Data

All continuous variables will be summarised using the following descriptive statistics: n (non-missing sample size), mean, standard deviation, median, maximum, and minimum. The frequency and percentages (based on the non-missing sample size) of observed levels will be reported for all categorical measures. All summary tables will be structured with a column for each treatment in the order (Placebo, Dexamethasone, Total).

## Subject Disposition

A CONSORT diagram (Figure 2) will be produced to show patient disposition. The number screened will be taken from screening logs. The number randomised will be calculated using the Tenalea generated patient ID number. The number excluded prior to randomisation will be calculated as the number screened minus the number randomised. The number excluded post-randomisation will be calculated as the number of ineligible patients in the MACRO database. The numbers allocated to dexamethasone or placebo will be found using the kit ID number and linking this to the concealment list which consists of the kit ID number and the treatment ID (taking values 1 or 2). The treatment ID will be replaced with the correct treatment labels following unblinding.

The number assessed at 3-months will be calculated as the number with a 3-month mRS score recorded (including those that have a score of 6 - Dead). The number withdrawn at 3-months will be calculated using the information on page 56 of the CRF (withdrawals from trial and follow-ups excluding deaths). Those patients that have no data at 3-months or 6-months will be classified as “lost to follow-up”. If a patient has 6-month data but no 3-month data then they will be classified as “transiently missing” at 3-months.

The number assessed at 6-months will be calculated as the number with a 6-month mRS score recorded (including those that have a score of 6 - Dead). The number withdrawn at 6-months will be calculated using the information on page 56 of the CRF. Those patients in whom no data is received at 6-months will be classified as “lost to follow-up”.

Figure 2: CONSORT diagram

##
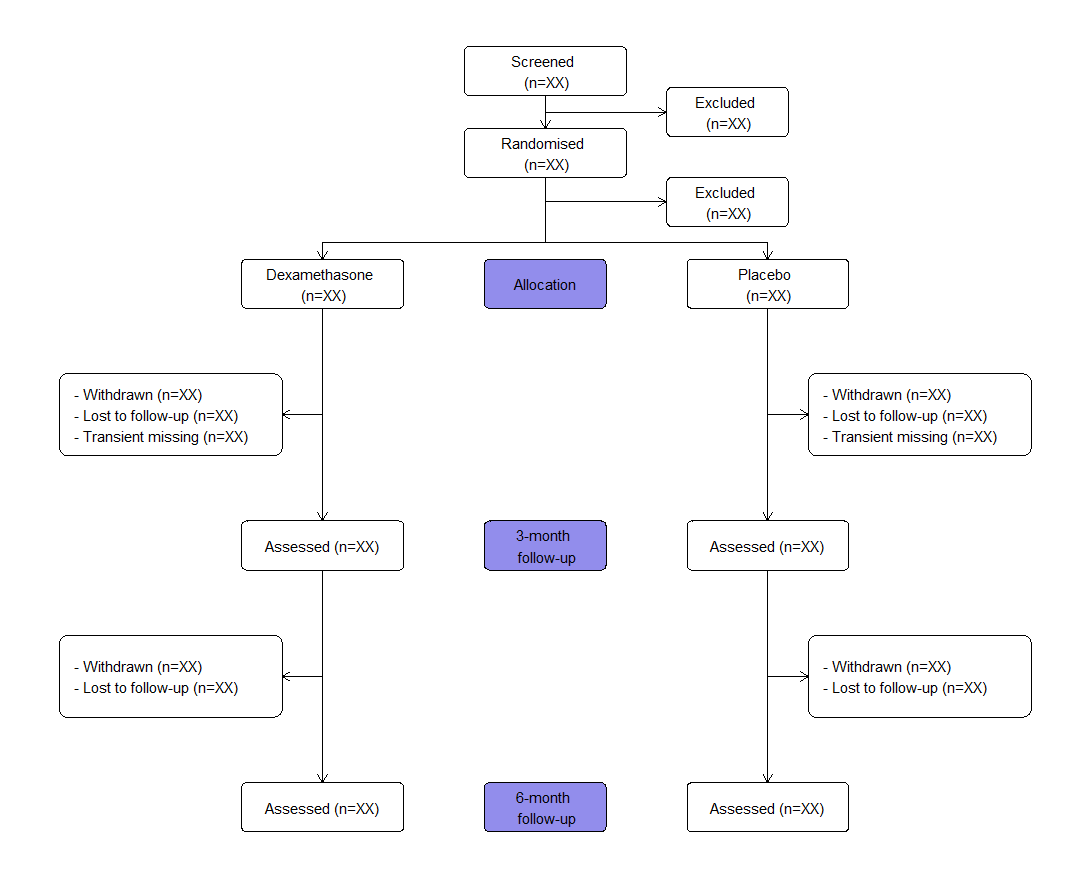


## Derived variables

- Patients that have died before completing the 3-month and/or 6-month mRS will be given an mRS score of 6
- The mRS binary outcome at all time-points will be categorised as either favourable (0-3) or unfavourable (4-6)
- GCS total scores will be categorised as 0 to 7, 8 to 12, and 13 to 15
- Burr holes will be categorised as one new burr hole (left, right, or bilateral), two new burr holes (left, right, or bilateral), and combination of one and two burr holes
- Length of stay in NSU (days) will be calculated as

$$\sum\text{\{(date of discharge or death) - (date of admission to NSU)\}}$$

where the summation is taken over all admissions

- Length of stay in intensive care/high dependency unit will be calculated as the total number of days in intensive care/high dependency unit across all admissions
- Length of stay in secondary care will be calculated as the length of stay in NSU plus the self-reported length of stay in hospital or healthcare facility based on the six month questionnaires
- Length of time in the trial (days) for withdrawals only will be calculated as the date of withdrawal minus the date of randomisation
- The proportion of medication taken will be calculated as

$$\frac{\text{Number of tablets taken across days 1 to 14}}{62}$$

- A new indicator variable will be created for centre where Cambridge = 1 and other sites = 0
- For subgroup analyses, age will be dichotomised to <70 and ≥70 years
- For subgroup analyses, a new variable for history of head trauma will be created taking the categories “no head trauma”, “head trauma ≤4 weeks ago”, “head trauma >4 weeks ago”, and “head trauma with unknown timing”
- For subgroup analyses, use of anti-coagulants will be defined as “yes” if a patient answers yes to taking any of the following: aspirin, clopidogrel, warfarin, or other anti-coagulant/anti-platelet, and will be defined as “no” otherwise
- For subgroup analyses, recurrent CSDH will be defined as “yes” for patients with ≥1 re-operation and “no” otherwise
- For subgroup analyses, conservative management will be defined as “yes” for patients that had no surgery during any admission and “no” otherwise
- For subgroup analyses, drain during index surgery will be defined as “yes” for those patients that had either a subdural or subgaleal drain during their primary surgery and “no” otherwise
- Percentage of medication taken will be calculated as

$$\frac{100\times\text{Total number of tablets taken}}{62}$$

## Protocol Deviations

Protocol deviations will be defined for both the placebo and dexamethasone groups as follows:

- Those patients who took <80% of their treatment (<50 tablets)
- Those patients whose assessments were outside the time-windows defined in Section 6.4

For the placebo group the following protocol deviation is also defined:

- Those patients that received >8mg of dexamethasone during the IMP course

Patients that deviate from the protocol based on the above definition will be excluded from the per protocol analysis populations.

## Demographic and Baseline Variables

The following demographic and baseline variables will be summarised according to Section 219 using the full analysis population.

### Demographics

- Age (years)
- Gender
- Race (Caucasian/White, Black, Asian, Hispanic, Other)

### Health Status Prior to CSDH

- Residence prior to CSDH (independent, carers at home, residential home, nursing home, other)
- Mobility prior to CSDH (independent, stick, zimmer frame, wheelchair, bed bound, other)
- Pre-morbid mRS

### Injury background

- Timing of onset of symptoms related to CSDH (<7 days, 7-14 days, 15-28 days, 29-42 days, >42 days, not known)
- Known head trauma (yes/no)
- If known head trauma, how long ago it occurred (<2 weeks ago, 2-4 weeks ago, 1-3 months ago, 4-6 months ago, >6 months ago, not known)
- mRS on admission to NSU
- GCS on admission to NSU

### Imaging, Surgery, and Complications

The following variables will be summarised:

- Side of CSDH (bilateral, left, right)
- Density of CSDH (hypodense, isodense, mixed density)
- Midline shift (0-5mm, >5 and ≤10mm, >10mm)
- Surgery number (no surgery, primary, 1st recurrence, 2^nd^ recurrence, 3^rd^ recurrence, other) - all admissions
- Surgery number - index admission
- Surgery number - subsequent admissions

The following variables will be summarised separately for primary and recurrent surgeries:

- Burr hole category (one burr hole, two burr holes, combination of one and two burr holes)
- Mini craniotomy category (bilateral, left, right)
- Burr hole reopened (bilateral, left, right)
- Burr hole converted to mini craniotomy (bilateral, left, right)
- Subdural drain inserted (bilateral, left, right)
- Subgaleal drain inserted (bilateral, left, right)
- Post-operative complications (post-op acute subdural haematoma (ASDH) operated, residual CSDH operated, post-op intracranial haemorrhage operated, tension pneumocephalus, seizure, surgical site infection, non-surgical site infection, VTE/DVT/PE, other)

Other post-operative complications will also be listed in a separate table for the primary and recurrent surgeries. Surgical variables (for example, burr hole category) will be summarised using frequency and percentages (based on the number of people that had surgery).

### Treatments Given

The following variables are binary yes/no responses:

- Vitamin K only
- Prothrombin Complex Concentrate (PCC) only
- Platelets only
- Fresh Frozen Plasma (FFP) only
- Packed Red Blood Cells (PRBCs) only
- Other only
- Combination of treatments

## Concurrent Illnesses and Medical Conditions

The following co-morbidities are binary yes/no variables and will be summarised according to Section 9 using the full analysis population:

- Diabetes
- Ischaemic heart disease
- Atrial fibrillation
- Metallic heart valve
- DVT/PE
- Stroke
- Previous CSDH
- Epilepsy
- Dementia
- COPD
- Liver disease
- Current malignancy
- Other

## Prior and Concurrent Medications

The following treatments taken prior to admission are binary yes/no variables and will be summarised according to Section 9 using the full analysis population:

- Anti-coagulants
  - Aspirin only
  - Clopidogrel only
  - Warfarin only
  - Other anti-coagulant only
  - Combination of anti-coagulants
- Other medications
  - Non-steroidal anti-inflammatory drugs
  - Diuretics
  - ACE inhibitors
  - Immunosuppressants
  - Antacids/Proton Pump Inhibitors

A separate table summarising the other anti-coagulant categories will also be produced.

## Treatment Compliance

The frequency and percentage of patients compliant at day 14 will be reported using the question “Were all 14 days of study medication taken per protocol?” - CRF page 41. Summary statistics for the proportion of medication taken over days 1 to 14 will be produced according to Section 9 for both compliant and non-compliant patients.

## Withdrawals

Listings of withdrawals from treatment only and withdrawals from the trial (ordered by withdrawal reason) will be produced and will consist of the following variables:

- Subject ID
- Site
- Treatment group
- Withdrawal reason
- Gender
- Age (years)
- Time in trial (days)

Summary statistics of the withdrawal reason (both for withdrawals from treatment only and withdrawals from the trial) will be produced according to Section 9 using the safety population.

## Recurrence

The frequency and percentage of recurrences (defined in Section 5.2.3) will be produced by treatment group for the subgroup of patients that undergo primary surgery, as by definition a subject cannot have a recurrence without undergoing primary surgery.

# Efficacy Analyses

The primary and secondary efficacy outcomes will be analysed using both the full analysis population and the per protocol populations.

## Primary Efficacy Analysis

The primary efficacy endpoint is

- mRS at 6 months which is dichotomised to favourable (0-3) vs unfavourable (4-6)

Summary statistics will be produced according to Section 9. The primary analysis will estimate the absolute difference between the two treatment arms in the proportions achieving a favourable outcome. A simple Normal approximation (z-test) will be used to produce a 95% confidence interval and two-sided P-value testing the following confirmatory hypotheses:

- Null hypothesis: there is no difference in the primary outcome between the two treatment arms
- Alternative hypothesis: there is a difference in the primary outcome between the two treatment arms

As a secondary analysis, a proportional odds logistic regression model will be fitted to the original mRS score adjusting for baseline covariates (age, GCS). The results from the proportional odds logistic regression model will be presented in a table showing the treatment effect (odds ratio - Dexamethasone versus Placebo), 95% confidence interval, and P-value.

To investigate how treatment compliance affects the primary outcome, the following exploratory analyses will be performed:

- Test for an interaction between treatment and the percentage of medication taken
- Calculate the complier average causal effect (CACE) for different cut-offs of compliance (>50%, >60%, >70%, >80%, >90%, 100% of medication taken) [14]
- Instrumental variables analysis to estimate the effect of compliance measured on a continuous percentage scale, where randomisation is the instrumental variable

The CACE estimation relies on the following assumptions:

- There are two latent classes of participants
  - Compliers get treatment if and only if randomly allocated to the treatment
  - Non-compliers never get the treatment regardless of allocation
- On average, the proportion of compliers is the same in the two arms of the trial as a result of randomisation
- For the non-compliers, the offer of treatment itself does not influence outcome
- There is an absence of defiers - patients that take the opposite treatment to what is offered

The results of this analysis may be biased as the latter condition may not hold. Whilst in general, non-compliers could be split into “never-takers” (as defined above) and “always-takers” (those patients that receive the active medication, dexamethasone, regardless of allocation), we assume that the probability of being an “always-taker” is zero, due to the fact that no patient in the placebo group will receive the full 14 day tapering course of dexamethasone.

## Secondary Efficacy Analyses

### Surgery

The surgery endpoints are:

- Number of CSDH related surgical interventions undertaken during the index admission
- Number of CSDH related surgical interventions undertaken during subsequent admissions in the follow-up period

The number of surgical interventions undertaken during the index admission will be defined in two-ways:

- Including pre-randomisation surgical procedures (which occur within 72 hours prior to randomisation)
- Excluding pre-randomisation surgical procedures

The analyses will be repeated for both definitions.

Summary statistics for the number of CSDH surgical interventions are described in Section 9.4.4. Poisson regression will be used to model the effect of treatment (Dexamethasone versus Placebo) on the outcomes. The results from the Poisson regression will be presented in a table showing the treatment effect, 95% confidence interval, and P-value. If the model is over-dispersed (i.e. the conditional variance exceeds the mean) then we will consider fitting a negative binomial model to the data instead.

### Questionnaires

The questionnaire endpoints are:

- GCS at discharge and 6-months
- mRS at discharge and 3-months
- BI at discharge, 3-months, and 6-months
- EQ-5D-5L at discharge, 3-months, and 6-months

Summary statistics for the GCS (individual questions and categorised total score), mRS (original and dichotomised scores), BI (individual questions and total score), and EQ-5D-5L (individual questions, VAS, and utility index) will be produced according to Section 9. A stacked bar chart of mRS outcomes by treatment group and assessment time-points (pre-morbid, admission, discharge, 3-months, 6-months) will be produced both including and excluding missing data.

For the GCS (categorised total score) and mRS outcomes (original score), proportional odds logistic regression will be used to model the effect of treatment (Dexamethasone versus Placebo) on the outcome. The results from the proportional odds logistic regression will be presented in a table showing the treatment effect (odds ratio - Dexamethasone versus Placebo), 95% confidence interval, and P-value.

For the BI (total score) and the EQ-5D-5L (utility index), linear regression will be used. The results from the linear regression will be presented in a table showing the treatment effect (Dexamethasone versus placebo), standard error, 95% confidence interval, and P-value.

### Mortality

The mortality outcomes are:

- All-cause mortality at 30 days and 6-months

A listing of deaths will be produced and will consist of the following variables:

- Subject ID
- Site
- Treatment group
- Gender
- Age (years)
- Time in trial (days)

A bar chart showing the number of deaths by key time-points (≤14 days, 15-30 days, 31-90 days, 90+ days) will also be produced. Logistic regression will be used to model the effect of treatment (Dexamethasone versus placebo) on the binary outcome of death (yes/no) at 30 days and 6-months. The results from the logistic regression will be presented in a table showing the treatment effect (odds ratio), 95% confidence interval, and P-value.

### Discharge Information

The discharge endpoints are:

- Length of stay in NSU (days)
- Length of stay in secondary care (days)
- Discharge destination from NSU (home - independent or with carers, local hospital, rehabilitation centre, residential home, nursing home, other)

Summary statistics will be produced according to Section 9. The frequency and percentage of patients that stayed in ICU/high dependency unit and their length of stay will also be summarised according to Section 9. Poisson regression will be used to model the effect of treatment (Dexamethasone versus placebo) on length of stay in NSU and length of stay in secondary care. The results from the Poisson regression will be presented in a table showing the treatment effect, 95% confidence interval, and P-value. If the model is over-dispersed (i.e. the conditional variance exceeds the mean) then we will consider fitting a negative binomial model to the data instead.

Logistic regression will be used to model the effect of treatment (Dexamethasone versus placebo) on discharge destination from NSU. The results from the logistic regression will be presented in a table showing the treatment effect (odds ratio), 95% confidence interval, and P-value.

### Health Economics Analyses

The health economics analyses will be performed by Professor Garry Barton and will be documented in a separate Health Economics Analysis Plan.

## Exploratory Efficacy Analyses

The exploratory outcomes were analysed by the PI (Ellie Edlmann) as part of their PhD.

# Safety Analyses

Listings of AEs will be ordered by treatment group and onset date. They will contain the following information:

- Subject ID
- Site
- Treatment group
- Event
- Onset date
- Resolution date
- MedDRA preferred term (PT)
- MedDRA system organ class (SOC)
- Causality
- Outcome
- Severity - for serious adverse events (SAEs) only
- Seriousness - for SAEs only
- SAE reference number - for SAEs only

The frequency and percentage of MedDRA SOC codes will be reported. All summary tables will be structured with a column for each treatment in the order (Placebo, Dexamethasone, Total). When calculating the incidence of MedDRA SOC codes, each subject will only be counted once and any repetitions will be ignored; the denominator will be the total population size. Plots showing the incidence of AEs and the relative risk (with 95% confidence interval) based on the MedDRA SOC codes will be produced. The analysis will be based on the safety population.

## Adverse Events of Special interest

The following adverse events are of special interest (AESI):

- Hyperglycaemia necessitating treatment
- Hyperglycaemia necessitating stopping of trial medication
- New onset diabetes necessitating on-going medical treatment at day 30 follow-up
- Hyperosmolar hyperglycaemic state
- New onset psychosis
- Upper gastrointestinal side effects (e.g. heartburn, vomiting)
- Peptic ulceration and gastro-intestinal bleeding

A listing of non-serious AESIs and summary statistics will be produced according to Section 11. A listing of serious AESIs and summary statistics will also be produced according to Section 11.

The following AESIs will also be summarised by past medical history of diabetes (yes/no) and treatment group:

- Hyperglycaemia necessitating treatment
- Hyperglycaemia necessitating stopping of trial medication

## Deaths, Serious Adverse Events and other Significant Adverse Events

SAEs are defined as any untoward medical occurrence or effect that:

- Results in death
- Is life-threatening
- Requires hospitalisation or prolongation of existing inpatients’ hospitalisation
- Results in persistent or significant disability or incapacity
- Is a congenital anomaly or birth defect
- Is another important medical event

The initial index surgery will not be reported as an SAE unless any of the above criteria are met, nor will SAEs be reportable if they are deemed due to complications related to the CSDH.

A listing of non-reportable SAEs and summary statistics will be produced according to Section 11. A listing of reportable SAEs and summary statistics will be produced according to Section 11. Reportable SAEs will also be summarised by outcome and will be presented split by timing of SAE as “early” if they occur within 30 days of starting the IMP and “late” if they occur after day 30.

## Pregnancies

Pregnancies will be presented as a listing consisting of subject ID, date of first study medication, and, if possible, date and outcome of pregnancy.

# Figures

The following figures will be produced:

- A CONSORT diagram showing patient disposition
- A stacked bar chart showing the mRS at each time-point (pre-morbid, admission, discharge, 3-months, 6-months)
- A bar chart showing the number of deaths by key time-points (≤14 days, 15-30 days, 31-90 days, 90+ days)
- A graph showing the percentage of missing data for each of the primary and secondary outcomes

# Reporting Conventions

P-values ≥0.001 will be reported to 3 decimal places; P-values <0.001 will be reported as “<0.001”. The mean, standard deviation, and any other statistics other than quantiles, will be reported to one decimal place greater than the original data. Quantiles, such as median, or minimum and maximum will use the same number of decimal places as the original data. Estimated parameters, not on the same scale as raw observations (e.g. regression coefficients) will be reported to 3 significant figures.

# Technical Details

The SAP is based on Version 3 of the protocol. The software package R version 3.5 will be used on a Windows computer and copies of the code written will be stored. Each report and individual table or graph will have:

- Date and time stamp
- Name of the code file used
- Author
- Population used

The version control system Git will be used and individual code files will also have comments that convey:

- Author
- Date and time of writing
- Description of any revisions
- Description of the inputs and outputs
- Reference to any parent code file that runs the code

A reviewing statistician will independently reproduce the following:

- mRS scores of 6 (Dead)
- The primary efficacy analysis
- Summary statistics tables 3.02 (Surgery information), 6.01 (Compliance information), and 7.01 (Discharge information)

They will have an overview of the entire analyses and explicitly check the code producing tables 3.01 (Injury background), 11.01 (EQ-5D at discharge), and 13.03 (AESIs by past medical history of diabetes).

# Summary of Changes to the Protocol

The tertiary outcome recurrence has been added as recurrence rate is reported in the majority of surgical CSDH studies, and calculation of this will therefore allow cross-study comparisons [14].

# References

| [1] | A. Bruno, A. Akinwuntan, C. Lin, B. Close, K. Davis, V. Baute, T. Aryal, D. Brooks, D. Hess and J. Switzer, “Simplified modified rankin scale questionnaire: reproducibility over the telephone and validation with quality of life,” *Stroke,* vol. 42, no. 8, pp. 2276-2279, 2011. |
| --- | --- |
| [2] | M. Herdman, C. Gudex, A. Lloyd, M. Janssen, P. Kind, D. Parkin, G. Bonsel and X. Badia, “Development and preliminary testing of the new five-level version of EQ-5D (EQ-5D-5L),” *Quality of Life Research,* vol. 20, no. 10, pp. 1727-1736, 2011. |
| [3] | B. van Hout, M. Janssen, Y. Feng, T. Kohlmann, J. Busschbach, D. Golicki, A. Lloyd, L. Scalone, P. Kind and A. Pickard, “Interim scoring for the EQ-5D-5L: mapping the EQ-5D-5L to EQ-5D-3L value sets,” *Value Health,* vol. 15, pp. 708-715, 2012. |
| [4] | National Insitute of Health and Care Excellence, “NICE position statement on the EQ-5D-5L,” 11 August 2017. [Online]. Available: https://euroqol.org/nice-position-statement-on-the-eq-5d-5l/. [Accessed 16 August 2018]. |
| [5] | G. Teasdale and B. Jennett, “Assessment of coma and impaired conciousness. A practical scale.,” *The Lancet,* vol. 2, no. 7872, pp. 81-84, 1974. |
| [6] | F. Mahoney and D. Barthel, “Functional evaluation: the Barthel Index: a simple index of independence useful in scoring improvement in the rehabilitation of the chronically ill,” *Maryland State Medical Journal,* vol. 14, pp. 56-61, 1965. |
| [7] | E. Houghton, L. Grainger and P. Teale, “The in vitro hydrolysis of dexamethasone trimethylacetate in whole blood from the horse and cow,” Unpublished, Newmarket, 1989. |
| [8] | A. Coert, M. Hoeijmakers and P. van Rens, “The in vitro hydrolysis of dexamethasone dimethylbutyrate in cows plasma,” Unpublished, Ingelheim, 1988. |
| [9] | T. Santarius, P. Kirkpatrick, D. Genesan, H. Chia, I. Jalloh, P. Smielewski, H. Richards, H. Marcus, R. Parker, S. Price, R. Kirollos, R. Pickard and P. Hutchinson, “Use of drains versus no drains after burr-hole evacuation of chronic subdural haematoma: a randomised controlled trial,” *The Lancet,* vol. 374, no. 9695, pp. 1067-1073, 2009. |
| [10] | T. Santarius, P. Kirkpatrick, A. Kolias and P. Hutchinson, “Working toward rational and evidence-based treatment of chronic subdural haematoma,” *ClinNeurosurg,* vol. 57, pp. 112-122, 2010. |
| [11] | P. Delgado Lopez, V. Martin Velasco, J. CastillaDiez, A. Rodriguez Salazar, A. GalachoHarriero and O. FernandexArconda, “Dexamethasone treatment in chronic subdural haematoma,” *Neurocirugia (Astur),* vol. 20, no. 4, pp. 346-359, 2009. |
| [12] | T. Sun, R. Boet and W. Poon, “Non-surgical primary treatment of chronic subdural haematoma: Preliminary results of using dexamethasone,” *Br J Neurosurg,* vol. 19, no. 4, pp. 327-333, 2005. |
| [13] | D. Jackson, I. White, D. Mason and S. Sutton, “A general method for handling missing binary outcome data in randomized controlled trials,” *Addiction,* vol. 109, no. 12, pp. 1986-93, 2014. |
| [14] | I. White, “Uses and limitations of randomization-based efficacy estimators,” *Statistical methods in medical research,* vol. 14, no. 4, pp. 327-347, 2005. |
| [15] | A. Chari, K. C. Hocking, E. Broughton, C. Turner, T. Santarius, P. J. Hutchinson and A. G. Kolias, “Core outcomes and common data elements in chronic subdural hematoma: a systematic review of the literature focusing on reported outcomes,” *Journal of Neurotrauma,* vol. 33, no. 13, pp. 1212-1219, 2016. |

# Listing of Tables, Listings and Figures

A listing of tables, listings, and figures can be found in the following document:

1. Patients on topical or inhaled steroids are allowed to be recruited into the trial, as are patients who have had one intra-operative dose of dexamethasone for anti-emesis [↑](#footnote-ref-1)
